# Sensitivity of mRNA Translation

**DOI:** 10.1038/srep12795

**Published:** 2015-08-04

**Authors:** Gilad Poker, Michael Margaliot, Tamir Tuller

**Affiliations:** 1 School of Elec. Eng.-Systems, Tel Aviv University, Israel; 2 Dept. of Biomedical Eng., Tel Aviv University, Israel

## Abstract

Using the dynamic mean-field approximation of the totally asymmetric simple exclusion process (TASEP), we investigate the effect of small changes in the initiation, elongation, and termination rates along the mRNA strand on the steady-state protein translation rate. We show that the sensitivity of mRNA translation is equal to the sensitivity of the maximal eigenvalue of a symmetric, nonnegative, tridiagonal, and irreducible matrix. This leads to new analytical results as well as efficient numerical schemes that are applicable for large-scale models. Our results show that in the usual endogenous case, when initiation is more rate-limiting than elongation, the sensitivity of the translation rate to small mutations rapidly increases towards the 5′ end of the ORF. When the initiation rate is high, as may be the case for highly expressed and/or heterologous optimized genes, the maximal sensitivity is with respect to the elongation rates at the middle of the mRNA strand. We also show that the maximal possible effect of a small increase/decrease in any of the rates along the mRNA is an increase/decrease of the same magnitude in the translation rate. These results are in agreement with previous molecular evolutionary and synthetic biology experimental studies.

During mRNA translation, complex molecular machines called ribosomes attach to the 5′ end of the messenger RNA (mRNA) and then scan it in a sequential manner. At each elongation step, a nucleotide triplet (codon) is read and the ribosome waits until a freely diffusing transfer RNA (tRNA), carrying the corresponding amino-acid, binds to the ribosome. The process ends when the ribosome reaches the 3′ end of the mRNA, detaches, and releases the chain of amino-acids that folds into a functioning protein^1^.

Translation is a crucial step in gene expression, and it is becoming increasingly clear that understanding this process is vital in order to reveal how biological systems develop, evolve, and function. Indeed, mRNA translation is the most extensively regulated step in mammals^2^, and a 100-fold range of translational efficiency was detected between different genes^3^,^4^. This clearly has a strong effect on the protein abundance that cannot be predicted by measuring mRNA abundances alone. In particular, two important dynamical aspects of the translation process are: (1) certain codons are “slower” than others due to factors such as low abundance of tRNA molecules with the corresponding anti-codon, folding of the mRNA, and interactions of the translated protein and the ribosome^5^; and (2) many ribosomes scan along the same mRNA chain in parallel and “traffic jams” can form behind a slowly moving ribosome.

TASEP is the standard mathematical model for translation^6^,^7^,^8^. In this model, particles move along a chain of *n* consecutive sites. Each site can be either occupied by a particle or free. A particle attaches to the first site with probability *α* (but only if this site is free), hops from site *i* to site *i* + 1 with probability *γ*_*i*_ (but only if site *i* + 1 is free), and hops from the last site of the chain with probability *β*. In the homogeneous TASEP, all the transitions rates *γ*_*i*_ are assumed to be equal and, without loss of generality, scaled to one. In the context of translation, the particles [chain] model the ribosomes [mRNA molecule].

The dynamic mean-field approximation of TASEP^9^, sometimes called the *ribosome flow model* (RFM)^10^, is a set of *n* ordinary differential equations:


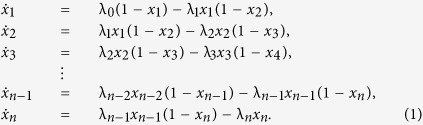


Here 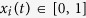 is the normalized occupancy level at site *i* at time *t*, and λ_*i*_ > 0 is a parameter that controls the transition rate from site *i* to the consecutive site *i* + 1. To explain this model, consider for example the equation


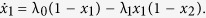


The term λ_0_(1 − *x*_1_) is the rate at which ribosomes attach to the beginning of the chain. This is given by the product of the initiation rate λ_0_ and the term (1 − *x*_1_). This means that as *x*_1_ increases, *i.e.*, as site 1 becomes fuller, the effective binding rate decreases. In particular, when *x*_1_(*t*) = 1 the site is completely full and the effective binding rate is zero. The term (1 − *x*_1_) thus reflects a “soft version” of the simple exclusion principle of TASEP. The term λ_1_*x*_1_(1 − *x*_2_) is the rate in which ribosomes move from site 1 to site 2. This is proportional to the occupancy level at site 1, and to (1 − *x*_2_) representing again the simple exclusion principle. The symmetry between the *x*_*i*_ and (1 − *x*_*i*_) terms also preserves the so called particle-hole symmetry of TASEP^11^. The term *R*(*t*): = λ_*n*_*x*_*n*_(*t*) ia the rate of ribosomes exiting from the last site, that is, the protein translation rate at time *t*.

Unlike TASEP, the RFM is a deterministic, synchronous, and continuous-time model. Nevertheless, it has been shown that for the range of parameters that are relevant for translation RFM and TASEP provide highly correlated predictions^10^.

The state-space of the RFM is the unit cube 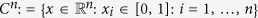. For 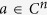, let *x*(*t*, *a*) denote the solution at time *t* of the RFM emanating from *x*(0) = *a*. For a set *S*, let int(*S*) denote the interior of *S*. It is known^12^,^13^ that the RFM admits a unique equilibrium 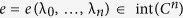, and that every trajectory of the RFM converges to *e*, that is, lim_*t*→∞_*x*(*t*, *a*) = *e* for all *a* ∈ *C*^*n*^. In particular, *R*(*t*) converges to the *steady-state protein translation rate R*: = λ_*n*_*e*_*n*_. In other words, the ribosomal densities and thus the translation rate always converge to steady-state values that depend on the rates, but not on the initial conditions.

Substituting *e* for *x* in (1) yields


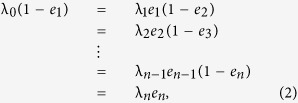


and this gives


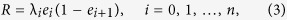


where we define 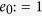 and 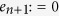. From this it is possible to obtain an elegant continued fraction (CF) equation for *R*. For example, for *n* = 2, (3) yields


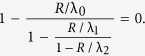


Solving the CF equation for *R* provides a mapping 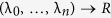 (note that since *e* ∈ *C*^*n*^ is unique, there is a single feasible solution *R*).

It has been recently shown^14^ that the (*n* + 2) × (*n* + 2) matrix:


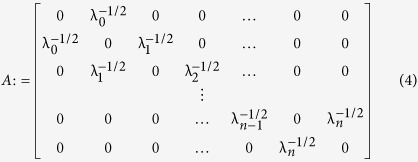


has real and distinct eigenvalues: 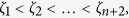 with


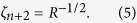


Furthermore, if we let *q*_*i*_, 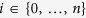, denote the (*i* + 1) × (*i* + 1) principal minor of (*ζ*_*n*+2_*I* − *A*) then


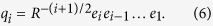


Equation (5) provides a *spectral representation* of the mapping from the λ_*i*_’s to *R*. As we will show below, this provides a useful framework for studying the dependence of the steady-state translation rate *R* in the RFM on the (generally inhomogeneous) initiation, elongation, and termination rates. Note that since *A* is a (componentwise) nonnegative matrix, *ζ*_*n*+2_ is also the Perron root^15^ of *A*, denoted *ρ*(*A*).

Note that the matrix *A* should not be confused with the transition matrix used in the stochastic analysis of TASEP, as that matrix decodes the transitions between all possible particle configurations and is thus of dimensions 2^*n*^ × 2^*n*^. This limits its use to very short TASEPs only.

Let 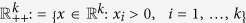. Recently, Eq. (5) has been applied^14^ to prove that the mapping 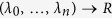 is strictly concave on 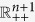. This means that the problem of maximizing *R*, subject to an affine constraint on the rates, is a *convex optimization problem*. Maximizing *R*, given the limited bimolecular budget, is important because it is known that translation is one of the most energy consuming processes in the cell^1^. Thus, it is natural to assume that evolution optimized this process. Also, maximizing the translation rate is a major challenge in synthetic biology and specifically in heterologous gene expression^16^. Several other tools from the field of systems and control theory have been used to analyze the RFM^13^,^17^,^18^.

## Results

**Figure 1:**
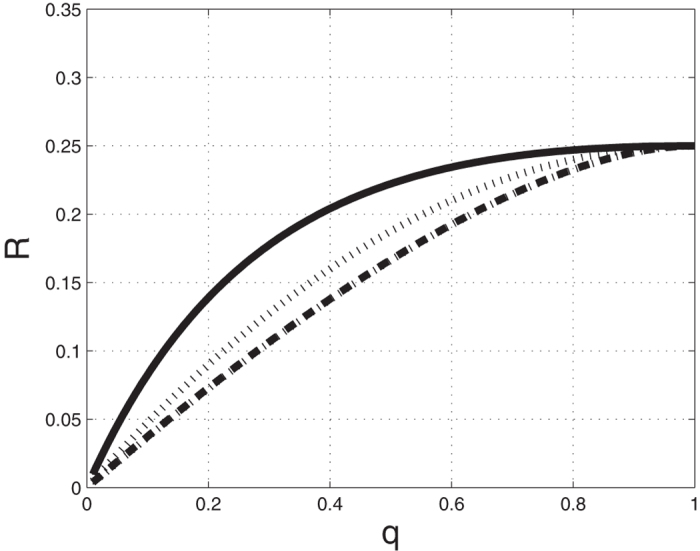
Steady state translation rate *R* in a RFM with *n* = 1000 and rates λ_*i*_ = 1, except for a configuration of slow rates with rate *q*. Solid line: λ_500_ = *q*; Dotted line: λ_500_ = λ_501_ = *q*; Dashed line: λ_499_ = λ_500_ = λ_501_ = *q*. As expected, *R* is an increasing function of *q*. Note that a cluster of consecutive slow sites considerably reduces *R*.

A number of interesting papers studied the effect of slow codon configurations on the steady-state translation rate using simulations and various approximations of TASEP^19^,^20^,^21^,^22^. The spectral representation of *R* provides a new, exact, and computationally efficient approach for studying this issue in the RFM using algorithms that compute the largest eigenvalue of a symmetric and tridiagonal matrix. For example, Fig. 1 shows *R*, computed via (5), for various slow rate configurations in an RFM with *n* = 1000.

Here, however, we use (5) to analyze a different (albeit related) notion, namely, the *sensitivities*:


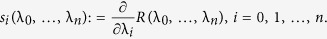


A relatively large value of *s*_*k*_ indicates that a small change in the rate λ_*k*_ will have a strong effect on the translation rate *R*. In other words, the sensitivities can be used to determine which rates are the most important in terms of their effect on the translation rate. The advantage of the spectral representation of *R* is that determining *s*_*i*_ becomes the classical eigenvalue sensitivity problem. Indeed, since *A* is (componentwise) nonnegative and irreducible^15^, there exists an eigenvector 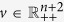 (that is unique up to scaling) such that *Av* = *ζ*_*n*+2_*v*. By known results from linear algebra^23^,


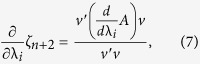


so


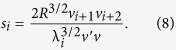


This provides a way to compute, in an efficient and numerically stable way, the sensitivities for large-scale inhomogeneous RFMs using standard algorithms for computing the eigenvalues and eigenvectors of symmetric tridiagonal matrices.

**Figure 2:**
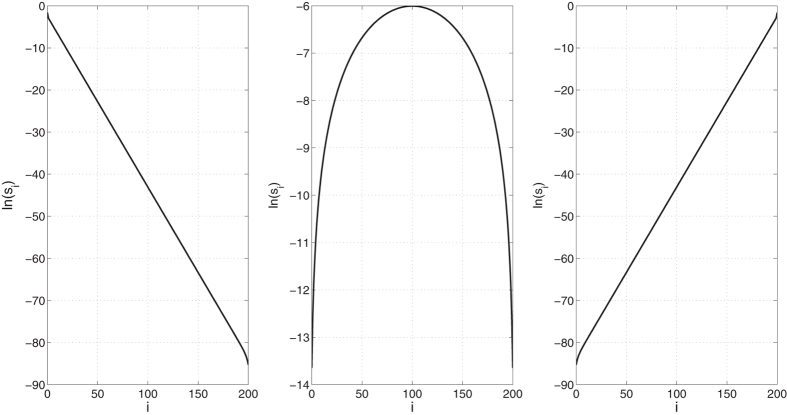
ln(*s*_*i*_) as a function of *i* in three HRFMs with length *n* = 200. Left: λ_0_ = 0.4 and λ_200_ = 1. Here λ_0_ is rate limiting and thus the sensitivities of sites close to site 1 are larger. Note that ln(*s*_*i*_) decays linearly with *i*. Middle: λ_0_ = λ_200_ = 1. Here the maximal sensitivity is with respect to λ_*n*/2_ and it decreases as we move towards the edges of the chain. Right: λ_0_ = 1 and λ_200_ = 0.4.

Figure 2 depicts ln(*s*_*i*_) as a function of *i* for three homogeneous RFMs (HRFMs) (*i.e.*, λ_*i*_ = 1 for all 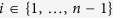), with size *n* = 200. The left sub-figure shows the case where λ_0_ = 0.4 and λ_200_ = 1, so λ_0_ is the rate limiting factor. The sensitivity *s*_0_ is maximal and the sensitivities decrease as *i* increases. This regime describes the typical case in endogenous genes where initiation is the rate limiting factor^24^. Such genes indeed demonstrate selection for increased robustness to transcription errors in ORF features that affect the translation rate (*e.g.* mRNA folding and adaptation to the tRNA pool)^5^. Similarly, our results may also explain the evolutionary selection for unusual codon usage bias at the ORF 5′ end^25^.

The right sub-figure shows the symmetric case where λ_0_ = 1 and λ_200_ = 0.4. The middle sub-figure depicts the case where *all* the λ_*i*_s are one. The plot demonstrates what is known in the TASEP literature as the *edge effect*^26^: the maximal sensitivity is in the center of the chain, and it decreases as we move toward the edges. This suggests that in order to maximize the translation rate in heterologous gene expression^16^, more attention should be devoted to tuning the codons in the middle of the coding sequence.

The spectral approach provides an upper bound on the sensitivities, namely,


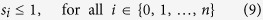


(see the Methods section for details). This means that the maximal possible effect of a small increase/decrease in any of the rates is an increase/decrease of the same magnitude in the translation rate. This agrees with a recent experimental study on the change in protein abundance resulting from perturbing the codons of heterologous genes 27. In this study, 25 variants of the viral gene *HRSVgp04* were generated and the corresponding protein levels were measured in *S. cerevisiae*. In each variant *only* codons 41–80 of the ORF were perturbed, without changing the encoded protein; thus, mRNA levels and translation initiation were expected to be identical in all variants. For each variant, the predicted change in the corresponding λ_*i*_s (*i.e.* transition rates) was computed^28^. An average change of 33.3% in the transition rate led to a 27.5% change in the protein levels.

We now focus on two special cases where it is possible to obtain *exact closed-form* expressions for the sensitivities.

### Totally homogeneous ribosome flow model (THRFM)

Suppose that λ_*i*_ = λ_*c*_ for *all* 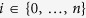. In other words, the initiation, termination, and all transition rates are equal, with λ_*c*_ denoting their common value. We refer to this case as the THRFM. In this case (see the Methods section)


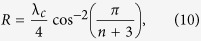


and the sensitivities are


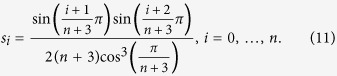


This provides a closed-form expression for the graph shown in the middle plot of Fig. 2.

Equation (5) implies that for any *c* > 0, 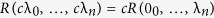. By Euler’s homogeneous function theorem, 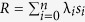, and for the THRFM this gives 
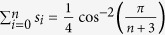
. Thus, 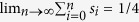.

**Figure 3:**
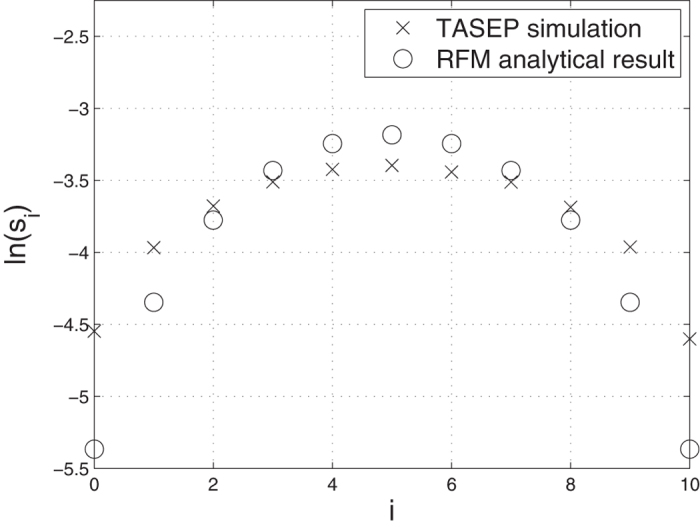
ln(*s*_*i*_) as a function of *i* for a THRFM with *n* = 10. The analytical results for the RFM (ο) are compared to TASEP sensitivity (x) calculated using Monte Carlo simulations.

Figure 3 compares the analytical formula (11) to simulations of the corresponding TASEP (see the methods section for details). It may be seen that the results agree reasonably well.

**Figure 4:**
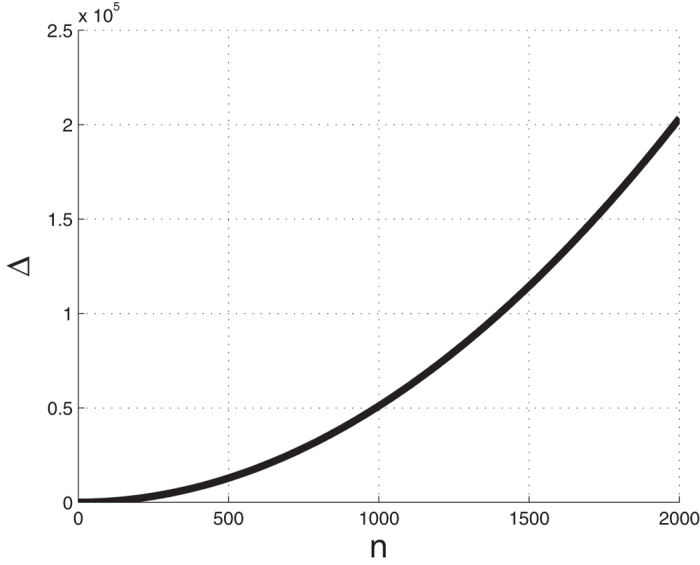
The function 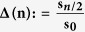 that measures the edge effect as a function of *n*.

An exact measure of the edge effect in the THRFM is given by the ratio 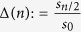. Substituting (11) and simplifying yields


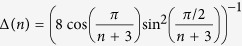


(see Fig. 4). Note that lim_*n*→∞_Δ(*n*) = ∞. In other words, although all the sensitivities decrease with *n* (see (11)) the edge effect actually becomes more prominent. One explanation for this edge effect is that a site at the center of the chain has more “close” neighbors than a site located towards one of the edges of the chain. Thus, when all the rates are equal the protein translation rate is more sensitive to the rates at the center of the chain.

### Homogeneous and symmetric ribosome flow model (HSRFM)

Another case where closed-form expressions for the sensitivities can be derived is when all the elongation rates are equal: 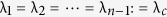, and also


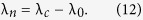


We refer to this case as the HSRFM. Here (see the Methods section for the details)


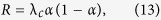


where 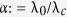. If *α* = 1/2 then


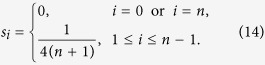


If 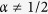,


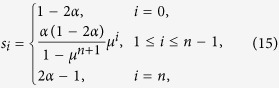


where 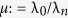. This can be explained as follows. If λ_0_ < λ_*n*_ (so *α* < 1/2 and *μ* < 1) then 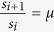, so ln(*s*_*i*+1_) − ln(*s*_*i*_) = ln(*μ*), for all 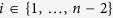. In other words, inside the chain the sensitivity strictly decreases with *i*, with ln(*s*_*i*_) decaying linearly with *i*. This is reasonable, as in this case the initiation rate is the limiting factor. Note that *s*_*n*_ < 0. This is due to (12), as increasing λ_*n*_ means decreasing λ_0_, and since this is the rate limiting factor, this decreases *R*. The case λ_0_ > λ_*n*_ is symmetric.

**Figure 5:**
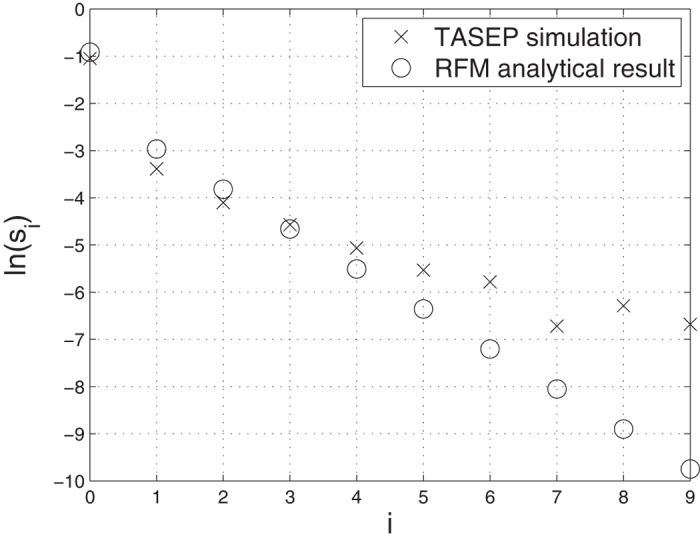
ln(*s*_*i*_) as a function of *i* for an HSRFM with *n* = 10, λ_*c*_ = 1 and λ_0_ = 0.3. The last value is not shown, as *s*_10_ = −*s*_0_ < 0. The analytical results for the RFM (ο) are compared to TASEP sensitivity (x) calculated using Monte Carlo simulations.

Figure 5 depicts ln(*s*_*i*_) in (15) for an HSRFM with *n* = 10, λ_*c*_ = 1, and λ_0_ = 0.3. The results are also compared to TASEP simulations (see the Methods section for details). It may be seen that the results agree with these simulations, except for large values of *i*, that probably require a larger number of simulations to obtain reliable averages. However, the sensitivity results for TASEP, obtained via Monte Carlo simulations, are problematic because the small change in the steady-state current, resulting from a small change in one of the transition rates, is masked by the inherent stochasticity of the process. Comparing Fig. 5 to Fig. 2 shows that the explicit equations for the HSRFM actually provide reasonable approximations to the general behavior of the HRFM in the case where the initiation rate is the limiting factor (and thus also in the symmetric case where termination rate is the limiting factor).

**Figure 6:**
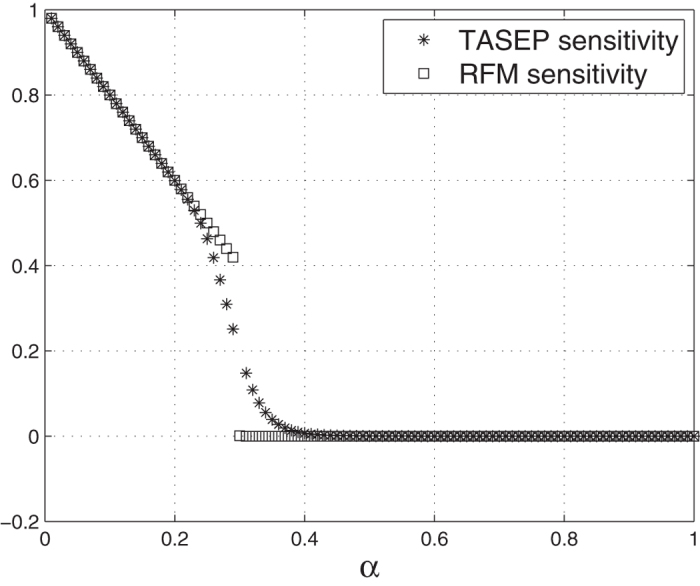
Sensitivity of the steady-state current *J* in the homogeneous TASEP (*) and of the corresponding homogenous RFM (◻) as a function of *α*.

For a more reliable comparison between the sensitivity of RFM and that of TASEP, we used the closed-form formula for the steady-state current in the *homogenous* TASEP with *N* sites given by *J* = Z(*N* − 1)/*Z*(*N*), where


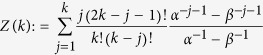


(see e.g. the survey paper^9^). Here *α* [*β*] is the entry [exit] probability and all the internal hopping rates *γ*_*i*_ are equal and normalized to one. We numerically calculated the sensitivity of *J* with respect to small changes in *α*, and compared this to the sensitivity of the corresponding RFM, i.e. the RFM with *n* = *N*, λ_0_ = *α*, λ_*i*_ = 1 ,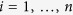, and λ_*n*_ = *β*. Figure 6 depicts 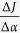 and 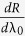 for the case *N* = *n* = 30, *β* = 0.3, and various values of *α*. It may be seen that the sensitivities agree well when 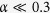 and when 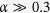. The value *α* = 0.3 corresponds to a phase transition in TASEP and then the sensitivity of the RFM, that is based on a mean-field approximation, differs from that of TASEP. In particular, it seems that the sensitivity near *α* = 0.3 in TASEP changes more smoothly than the sensitivity in the corresponding RFM.

## Discussion

Steady-state properties of the dynamic mean-field approximation of TASEP can be represented in a spectral form. Using this, we studied the sensitivity of the steady-state translation rate to perturbations in the initiation, transition, and termination rates. In this context, the problem reduces to the sensitivity analysis of the Perron root of a symmetric, nonnegative, tridiagonal, and irreducible matrix. This leads to: (1) highly efficient numerical computation of the sensitivities that is thus applicable for large-scale inhomogeneous RFMs; and (2) exact, closed-form expressions for the sensitivities in some special, yet important, cases.

Our results are based on analyzing the RFM which is the dynamic mean-field approximation of TASEP. However, simulations suggest that these results agree with the sensitivities in TASEP, which is the standard model for mRNA translation. The correspondence between the two models breaks down at parameter values that correspond to a phase transition in TASEP. In the remainder of this section, we discuss the relationship between the analytical results for the RFM sensitivity and known experimental findings, and suggest some future research directions.

First, a typical regime in the case of endogenous genes is when the initiation rate is the bottleneck rate^1^. The analytical results imply that in this case mutations at the 5′ end of the ORF will have a higher effect on the translation rate than mutations downstream of this region. The error rate in the process of gene transcription is estimated to be 1 in every 10^4^ nucleotides^1^. Thus, on average, one in every 67 windows with a length of 50 codons includes an error. Considering the fact that there are thousands of copies of mRNA molecules in the cell (for example, the number of mRNA molecules in *S. cerevisiae* [E. coli] is around 60000^29^ [1380]^30^), and that genes are transcribed and translated continuously suggests that the probability of an error that reduces the organism’s fitness is non-negligible. Thus, evolution should shape this region such that transcriptional mutations will have a lower effect on translation rates than in the rest of the ORF. Indeed, a recent study has shown that this may be the case^5^. This study included a computational analysis of all 40 nucleotide windows in endogenous ORFs in two model organisms: the prokaryote *E. coli* and the eukaryote *S. cerevisiae*. For each window, and for each coding sequence the effect of all possible point transcriptional mutations on features known to affect translation elongation rate (*e.g.* adaptation of codons to the tRNA pool^28^ and local strength of mRNA folding^5^,^25^) was computed; such a profile of each gene is actually a prediction of the mean effect of mutations on the local nominal translation rate (i.e. the different λ_*i*_ for all the sites *i* in our model) for that gene. At the next step, the mean of this profile over all the coding sequences of the organisms was computed to obtain the genomic profile of robustness of nominal translation rate to mutations (transcription errors). It was found that the robustness to mutations is higher at the beginning of the coding sequences (i.e. mutations at the beginning of the ORF tend to have a lower effect on the nominal translation rate). This suggests that there an increased selection for robustness to the effect of mutations on translation rate at the beginning of genes. This result agrees with the current study: if sensitivity of mRNA translation to small changes in λ_*i*_ is higher at the beginning of the coding sequence it makes sense that evolution shaped these regions such that the effect of mutations on λ_*i*_ will be lower.

Second, another study supports our analytical results which imply that mutations (small changes in λ_*i*_) at the 5′ end of the ORF will have a higher effect on the translation rate than mutations downstream of this region. A recent synthetic biology experiment (under review) estimated the distinct and causal effects of different parts of the transcript in the eukaryote *S. cerevisiae*. Reporter libraries of the viral *HRSVgp*04 gene have been generated for studying the effect of mutations in codons 2–41 and codons 42–81 of the ORF on translation rate and thus protein levels. The first library, named *L*2–41, measured the protein levels of various variants of different codons in positions 2–41 (i.e. different λ_*i*_) but with the same codons elsewhere. The second library, *L*42–81, measured the protein levels of various variants of different codons in positions 42–81 but with the same codons elsewhere. It is important to mention that the size of these regions is close to the typical chunk corresponding to a single state-variable in the RFM^10^ (around 25 codons). It was found that the variability in protein levels is higher in the first library than in the second one; this suggests that the λ_*i*_s in the first codons have a higher effect on translation rate, supporting the analysis reported here.

Third, we show that in the RFM the maximal possible effect of a small increase/decrease in any of the elongation rates along the mRNA is an increase/decrease of the same magnitude in the translation rate. Using library *L*42–81 described above, it was possible to estimate the expected effect on translation elongation rate via applying a novel measure of per codon elongation rate^28^ (corresponding to the λ_*i*_s in the RFM) which is based on local ribosome density measurements using ribo-seq experiments^31^. Average changes of 33% in the rate led to an actual 27% average effect on the measured protein levels. This agrees well with our bound *s*_*i*_ ≤ 1, suggesting that *s*_*i*_ can be helpful for designing synthetic genes, as it provides an estimate of the effect of synonymous mutations on the resulting protein levels.

Finally, we show that when all the rates are more or less equal, as may be the case for highly expressed and/or heterologous optimized genes, the maximal sensitivity is at the middle of the mRNA sequence. A common biotechnological approach for protein production is heterologous gene expression^16^,^32^ in which a target protein is expressed in a host cell. Often, the corresponding gene and host cell are optimized in order to maximize the protein levels of the target protein. In such cases, it makes sense to maximize all gene expression steps, and to make all of them uniformly efficient such that there are no bottlenecks. Furthermore, it also makes sense to design the heterologous gene such that these features will be maintained under different perturbations of the intracellular gene expression machinery. Our results suggest that perturbations near the middle of the ORF are expected to have a more prominent effect on the translation rate; this fact should be taken into consideration when designing heterologous genes.

It is important to mention that the conclusions reported here may also be relevant for other intracellular processes such as transcription of RNA, DNA replication, or intracellular trafficking over microtubule^1^ that involve macro-molecules movement over a polymer. For example, transcription has been modeled using a mathematical model that is similar to the RFM^33^ (in this case, the model describes the movement of RNA polymerase instead of ribosomes), and TASEP has posed the grounds for the development of theoretical tools describing molecular motors, with a good qualitative agreement when compared to state of the art experiments^34^. We believe that the approach reported here should have practical contributions to the study of gene expression in various biomedical disciplines including evolutionary biology, functional genomics, and synthetic biology.

An interesting research topic is based on further validating and studying the results reported here both computationally and experimentally. Experiments similar to the ones described above where genes with different initiation rate and (constant) elongation rate should be engineered and the effect on translation rate (or protein levels) of small changes in the decoding rates in different parts of the coding sequence should be measured. Comprehensive computational models of gene expression that may include additional aspects that are not included in the RFM (e.g. mRNA degradation, the stochastic nature of the process, the fact that it may not converge to a steady state, the size of ribosomes, frame shifts) can be employed to understand the effect of small changes in the elongation rates on the translation rate and protein levels *in vivo*.

## Methods

### Derivation of Eq. (9)

Pick 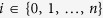. Since *v*_*k*_ > 0 for all 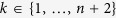, Eq. (8) implies that *s*_*i*_ > 0, *i.e.* an increase in any of the rates increases the steady-state translation rate. To determine an upper bound on *s*_*i*_, perturb λ_*i*_ to 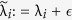, with 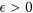. This yields a perturbed matrix 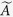 that is identical to *A* except for entries (*i* + 1, *i* + 2) and (*i* + 2, *i* + 1) that are:


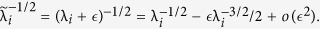


Thus, 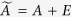, where *E* is a matrix with zero entries except for entries (*i* + 1, *i* + 2) and (*i* + 2, *i* + 1) that are 
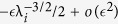
. By Weyl’s inequality^15^, 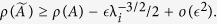 This yields 
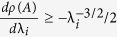
, so 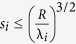. Since *R* ≤ λ_*i*_ for all *i*^10^, this yields (9).

### Derivation of the sensitivities in the THRFM

For the THRFM, the matrix *A* in (4) becomes 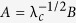, where 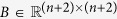 is a tridiagonal Toeplitz matrix with zeros on the main diagonal, and ones on the super- and sub-diagonal. It is well-known^35^ that the Perron root and Perron eigenvector of *B* are 
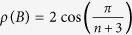
, and 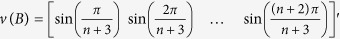. Therefore, 
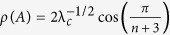
, and using *R* = *ρ*^−2^(*A*) yields (10). Substituting these values in (8) and using the fact that 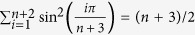 yields (11).

### Derivation of the sensitivities in the HSRFM

In the HSRFM, the matrix *A* in (4) becomes 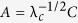, where


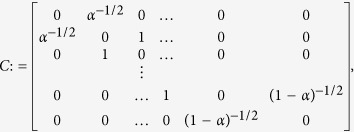


with 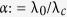. For *α* < 1, *ρ*(*C*) = (*α*(1 − *α*))^−1/2^, and *v*(*C*) is given by


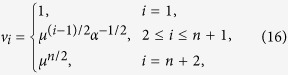


where 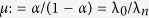. Indeed, a calculation shows that *Cv* = *ρv*. Thus, *v* is an eigenvector corresponding to the eigenvalue *ρ*, and since 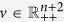, this eigenvalue is the spectral radius. Note that if *α* = 1/2 then 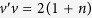, and otherwise 
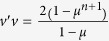
. Thus, *R* = *ρ*^−2^(*A*) = λ_*c*_*α*(1 − *α*). The sensitivities in the HSRFM can now be determined. Here *s*_*i*_, 1 ≤ *i* ≤ *n* − 1, were calculated using (8). Due to the additional coupling in (12), *s*_0_ and *s*_*n*_ cannot be computed using (8), so we used (7). This yields (14) and (15).

### TASEP Simulation

Every site *i*, 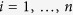, in the TASEP chain is associated with “hopping-times”, where the time between two consecutive hopping times is exponentially distributed with rate λ_*i*_. Hopping times into the first site are distributed with rate λ_0_. A simulation begins with an empty chain, and continues for 10^8^ time steps. At each step all sites are scanned. If site *i*'s hopping time is equal to the simulation time, then the next hopping time is calculated. Also, if site *i* is occupied and site *i* + 1 is free, the particle hops from site *i* to site *i* + 1. The steady-state average occupancy of each site is the number of time steps it was occupied divided by the total simulation time, with the first 10^6^ steps excluded from the calculation. For sensitivity simulations, TASEP with *n* = 10 sites is simulated 11 times. At each simulation, a different rate λ_*i*_ is perturbed by 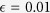, while the rest of the rates are left unchanged. Sensitivity for this iteration is then calculated as Δ*J*/Δλ_*i*_ where 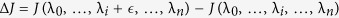 is the change in the average steady-state current, and 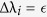. Results are averaged over 1000 iterations. As noted above, these results must be taken with care, as the small change in *J* corresponding to the 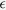 perturbation may be masked by the inherent stochasticity of the process. This is why we also added a comparison to the analytic closed-form for *J* available for the *homogeneous* TASEP. The MATALB code for the simulations is available in the supplementary material accompanying this paper (and can be downloaded from: http://www.cs.tau.ac.il/:tamirtul/supp_material_code.zip).

## Additional Information

**How to cite this article**: Poker, G. *et al.* Sensitivity of mRNA Translation. *Sci. Rep.* **5**, 12795; doi: 10.1038/srep12795 (2015).
